# Severe Hemoperitoneum after Patient Self-Induced Fecal Evacuation

**DOI:** 10.1155/2011/313841

**Published:** 2011-08-24

**Authors:** S. Gianesini, S. Lanzara, R. Stano, S. Santini, A. De Troia, S. Gennari, G. Vasquez

**Affiliations:** General Surgery Department, Emergency Surgery Service, University of Ferrara, Corso Giovecca 203, 44100 Ferrara, Italy

## Abstract

An increasing incidence of rectal injuries following patient self-induced harmful acts, aimed to sexual or laxatives porpouses, is a fact reported in literature (El-Ashaal et al., 2008). We herein report a case of severe hemoperitoneum related to a middle and upper rectal third seromuscolar tear caused by a self-induced fecal evacuation by means of an arrow with a covered cork tip. An urgent intestinal diversion by means of a Hartmann's operation was performed. The clinical case is presented in relation to the literature debate, regarding the issue of primary repair or resection and anastomosis versus fecal diversion for penetrating rectal injuries (Fabian, 2002; Cleary et al., 2006; Office of the Surgeon General, 1943; Busic et al., 2002). In conclusion, the importance of avoiding an anastomotic breakdown in a patient undergoing a hemorrhagic shock is highlighted.

## 1. Background

An increasing incidence of rectal injuries following patient self-induced harmful acts, aimed to sexual or laxatives purposes, is a fact reported in the literature [1].

We herein report a case of severe hemoperitoneum after a self-induced fecal evacuation, which requested an urgent intestinal diversion by means of a Hartmann's operation.

The clinical case is presented in relation to the literature debate, regarding the issue of primary repair or resection and anastomosis versus fecal diversion for penetrating rectal injuries [2–5].

## 2. Case Presentation

At 00.15 am a 69-years-old man was referred to our General Surgery Department for abdominal pain in the lower quadrants, consequent to a patient-referred self-induced manual fecal evacuation followed by a lypothymia event occurred 2 hours before.

The pathological anamnesis recorded arterial hypertension, prostatic hypertrophy, chronic gastritis, colic diverticulosis, and stypsis. Anticoagulants or platelet-inhibiting drugs were never assumed by the subject, who showed a normal coagulation profile. 

The abdominal pain was described by the patient as a dull ache with a sudden onset in the left iliac fossa, fastly affecting also the right inferior quadrant. No nausea, vomiting, or fever were associated. 

The physical examination revealed a mild diffuse abdominal distension; gently and deep palpation were feasible but mildly pain-evoking in the lower quadrants, where the Blumberg sign was considered slightly positive; the percussion revealed tympany in all the abdominal quadrants with the exception of the left iliac fossa were a dullness was recorded; the auscultation was silent.

The digital rectal examination assessed neither blood traces nor feces. The patient was hemodynamically stable without diuresis contraction. The emergency lab test showed a decreased in the haemoglobin level from 9.5 gr/dL (hematocrit 28%) to 7 gr/dL (hematocrit 21%) in the first 2 hours from the hospital admittance.

The plain abdominal X-rays just revealed some colic coprostasis in absence of perforation signs.

**Figure 1:**
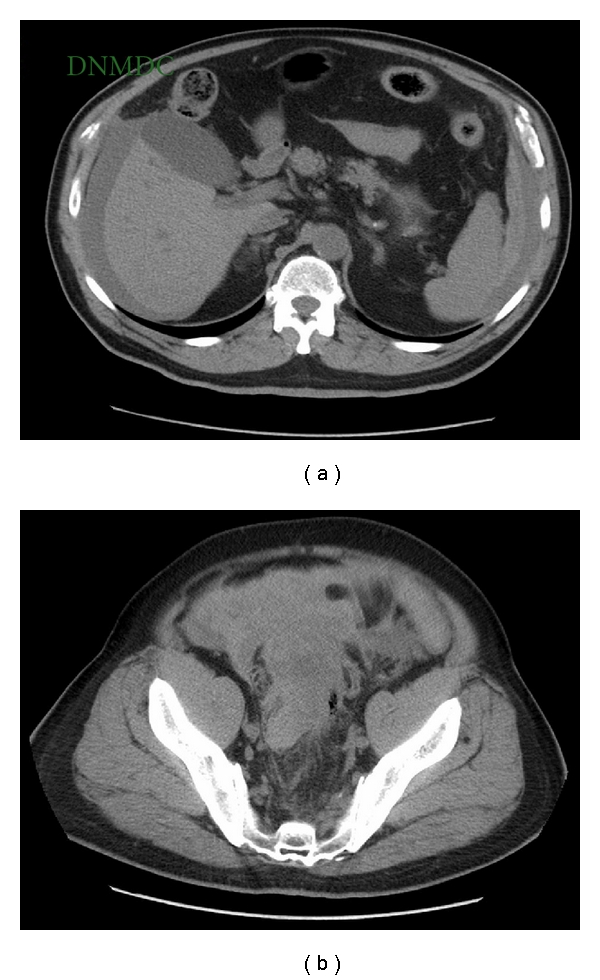
Preoperative computed tomography scan of the abdomen showing extensive hemoperitoneum (a) below the spleen and liver, among the small bowel, (b) in the left and right paracolic gutter, in front of the urinary bladder with a distended rectum.

Abdominal ultrasonography assessed free intraperitoneal fluid with imbibition of extraperitoneal structures, particularly in the lower right abdomen, while the CT scan with contrast media injection (Figure 1) highlighted a prevalent extraperitoneal area of enhanced dense fluid with contrast extravasation embracing the ciecum and sigmoid colon, associated with fluid of the same density below the spleen and liver and among the small bowel. 

The selective angiography was negative. Taking in consideration the hemoglobin drop and the instrumental results, a 2 international units blood transfusion and an urgent exploratory laparotomy were considered mandatory. 

In the operation field, an extensive hemoperitoneum was revealed (2.5 litres).

**Figure 2:**
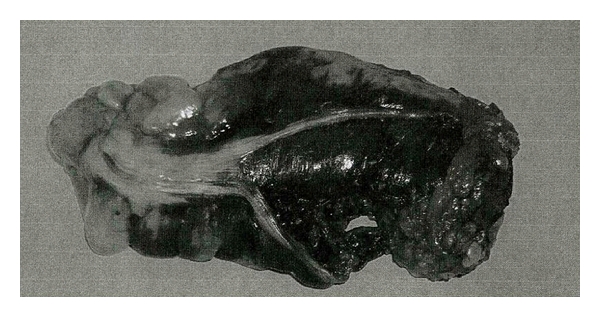
Surgical view shows an edematous rectal wall with hemorrhagic infiltration of the upper and medial rectal third. A seromuscular tear in the anterior tenia of the rectum is evident, together with an intact, bulging mucosa.

The rectum was edematous with hemorrhagic infiltration. The subsequent intramural hematoma extended along the middle and upper rectal third, determining a 7 cm long seromuscular tear in the anterior tenia (Figure 2), responsible for the massive intra-abdominal bleeding.

The patient required a Hartmann's operation owing to edematous and infiltrated colonic wall of questionable viability.

The patient's postoperative course did not report any minor or major complications, and he was discharged on postoperative day 8.

After 7 months, neither major nor minor complications were reported, so the patient was successfully submitted to a colostomy reversal by means of a traditional colorectal continuity restoration in accordance with the second step of the procedure originally described by H. Hartmann.

Considering the operative findings not matching with a complication of a self-induced manual fecal evacuation, as preoperatively reported by the patient himself, a more detailed questioning to him revealed his own personal use of an arrow with a covered cork tip to obtain relief from his chronic stypsis.

## 3. Discussion

Even already present in the past decades [5], the issue of primary repair or resection and anastomosis versus fecal diversion is still a main topic of debate, and certainly, it is worthy of further study: 10% to 15% of patients with traumatic intestinal injuries develop major infectious complications of which 30% fatal [2].

Guidelines for the management of colorectal injuries were developed by the Eastern Association for the Surgery of Trauma (EAST) Practice Parameter Workgroup [6]. 

Those who advocate fecal diversion believe that the incidence of septic complications is less present or have shown that the incidence of stoma closure is associated with acceptable morbidity [3].

Moreover, they cite the theoretical risk for abscess and necrotizing soft-tissue infections of the pararectal fat [3].

Levine et al. [7] in a review with 30 patients with extraperitoneal rectal injuries suggested that primary repair can be performed whenever in absence of any other major injuries until the first 8 hours. 

In the lack of a total international literature consensus about such procedure, we found adequate to treat the reported injury by means of a temporary diversion because of the edematous and infiltrated colonic wall of questionable viability. Thus, we chose to avoid the risk of anastomotic breakdown in a patient undergoing a hemorrhagic shock.

Certain histological intestines characteristics explain the peculiar manifestation of a rectal hematoma: it is the high tensile submucosa strength that protects the soft mucosa from shearing and stretching forces, while it is the loose attachment of the muscularis to the submucosa that permits formation of a cleavage plane at this junction when subjected to traumatic forces [8]. The final morphological feature is that intramural hematomas expand, causing dissection of the anterior teniae coli with subsequent rupture into the peritoneal cavity [9].

Progression of an intramural hematoma can lead to intestinal obstruction, perforation, and seromuscular tear with massive intra-abdominal bleeding: an acute clinical awareness is than requested. 

Nowadays, multiple imaging modalities improve acute lower intestinal bleeding diagnostic accuracy although with the different reported sensitivities: scintigraphy (93%) [10], catheter-directed angiography (40–86%) [11], endoscopy (70%) [12], and CT angiography (90.9%) [13].

The herein presented acute lower intestinal bleeding highlighted the usefulness of a CT evaluation, realistically justifying the negative angiographic assessment with a bleeding rate lower than 0.5 mL/min.

## 4. Conclusions

Of course, we believe that a conservative approach can be assumed first, but we consider surgery mandatory as soon as progressive enlargement of the intramural hematoma, intra-abdominal hemorrhage, generalized peritonitis, or intestinal obstruction develop.
